# Non-suicidal self-injury in inpatient and outpatient adolescents: disentangling psychopathology and interactive family dynamics

**DOI:** 10.3389/fpsyt.2024.1483745

**Published:** 2025-01-10

**Authors:** Rachele Fasolato, Alessia Raffagnato, Marina Miscioscia, Michela Gatta

**Affiliations:** ^1^ Department of Neurosciences, Biomedicine and Movement Sciences, University of Verona, Verona, Italy; ^2^ Department of Women's and Children's Health, School of Medicine and Surgery, University of Padua, Padua, Italy; ^3^ Child Neuropsychiatry Unit, University Hospital of Padua, Padua, Italy; ^4^ Department of Developmental and Social Psychology, University of Padua, Padova, Italy

**Keywords:** non-suicidal self-injury, psychopathology, adolescents, inpatients, outpatients, family dynamics

## Abstract

**Background:**

Non-suicidal self-injury (NSSI) is defined as a transdiagnostic phenomenon that has well increased in the latest years, especially in the adolescent population. It has been associated with suicidality, alexithymia, emotion dysregulation, and psychosocial impairment, as well as family issues. The choice of level of care (i.e., hospitalization versus outpatient visit) depends on a number of factors that relate not only to suicidal risk but also to severity of individual’s psychosocial functioning, the ability of family environment to support treatment choices and to contain child, as well as the need for ongoing monitoring of the young patient. A scarcity of studies has compared outpatients with inpatients, both of them engaging in NSSI.

**Methods:**

the current study aimed to further expand knowledge regarding features that characterize young self-harmers who receive different levels of care, with particular attention on psychopathological, family, and NSSI-related characteristics, as well as suicidality. The current research included 56 inpatients and 56 outpatients with NSSI, paired for gender, age, and psychiatric diagnosis. Instruments investigating psychopathology, emotion dysregulation, alexithymia, psychosocial functioning, and interactive family dynamics were administered. Descriptive statistics, parametric and non-parametric inferential statistics were applied.

**Results:**

study findings highlighted that inpatients engaging in NSSI reported lifetime suicidality, clinical level of externalizing and internalizing problems, more severe alexithymia, emotion dysregulation, and impaired psychosocial functioning compared to outpatients engaging in self-harming. Furthermore, when compared to outpatients’ families, inpatients’ families were more capable of adhering to rules and time of the family play situation (the Lausanne Trilogue Play procedure) and fixing interactive mistakes through activities. On the contrary, in the inpatient group, global performance, role implication, parental scaffolding, child’s involvement and self regulation tend to decline, while parental conflicts tend to rise over the four part scenario of the family play.

**Conclusion:**

these findings confirmed a more severe global picture of young inpatients engaging in self-harming, suggesting that NSSI may be the expression of this larger psychopathological picture. In addition, the study highlighted the need for a multi-informant and multimethod clinical assessment, which should include evaluation of family context and co-parenting system, especially for hospitalized young patients engaging in self-harm.

## 1 Introduction

Non-suicidal self-injury (NSSI) is defined as behavior of intentional harm to one’s body tissue in the absence of a suicidal purpose (1), although several studies have identified frequent coexistence between NSSI and suicidal behavior (2, 3). It is a transdiagnostic phenomenon; in fact, it is associated with a wide variety of psychiatric disorders in both outpatient and inpatient samples (4–7). Research showed a NSSI prevalence in nonclinical adolescent populations of 20% (8). This tends to rise in clinical populations to over 50% (5, 9). A further increase in NSSI among adolescents has been recorded during the Covid-19 pandemic (10, 11). The NSSI methods can be multiple (12), such as “cutting,” “scratching,” “head banging,” “burning,” and “hitting” (13, 14).

Nock and Pristein (2004) proposed a model that conceptualizes the different functions of NSSI along two axes: the positive/negative reinforcement axis and the intrapersonal/interpersonal axis (15). Subsequent research has shown that the one most reported is the intrapersonal function (16) in both inpatients (17) and outpatients (18). Unlike interpersonal function, intrapersonal function also positively predicts the severity of NSSI in terms of method versatility and NSSI frequency (19). The severity of the clinical picture has also been associated with more injured body sites or an injury in a body site other than the arms (20). Non suicidal self-injurers often reported greater psychosocial impairment (21, 22) and alexithymia, which is the difficulty in identifying and describing emotions (7, 23, 24). Moreover, Andover and Morris (2014) showed an association between NSSI and a low ability to regulate emotions adaptively (25). As a consequence, NSSI can be used as a dysfunctional coping mode that allows avoidance or reduction of negative emotions (15, 26).

The literature has also investigated the social and family context associated with NSSI, identifying dysfunctional family dynamics (27, 28), particularly in terms of the emotional dimension. A history of emotional abuse (29), a lack of parental emotional validation, a prevalence of negative affect and control (30), more rigid punitive methods, and an insecure parent-child attachment relationship (31) have been found. In addition, Halstead and colleagues (2014) reported poor family cohesion and communication issues in these families (32), whereas other authors found high family conflict (33, 34), low parental monitoring (34), and poor relationships with caregivers (33). A study conducted by Wang and colleagues (2022) showed a mediating effect of depressive symptoms on the relationship between poor family functioning and NSSI (35). Specifically, it has been suggested that the onset of depressive symptoms within a dysfunctional family context might be linked to the low development of the child’s ability to regulate emotions (36).

Emergency Department (ED) presentation for self-harm has increased in the last years, and this trend has accelerated, most of all in young females, since the Covid-19 outbreak (37). Nevertheless, a systematic review and meta-analysis conducted by Witt and colleagues (2023) showed that just one-in-five adults and young people who were referred for inpatient treatment were admitted following hospital presentation for self-harm, whereas just over half of young people referred for outpatient treatment after hospital presentation for self-harm attended at least one treatment session (38). In addition, Bridge and colleagues (2019) noted that only about one in four cases resulted in an outpatient follow-up appointment being scheduled before the patient leaves the emergency room (39), despite non-suicidal self-injury — especially if it occurs repeatedly over time — is thought to be a precursor of suicidal behavior (40). Moreover, the time after hospital discharge is considered to be the peak risk period for both non-suicidal self-harm and suicide (38). The choice regarding the level of care (hospitalization versus outpatient visit), in turn, depends on a number of factors that relate not only to suicidal risk (i.e., previous history of suicidal behaviors, depressive disorders, and high-lethality self-harm), but also to the severity of the individual’s psychosocial functioning, the ability of the family environment to adhere to and support treatment choices, and the need for ongoing monitoring of the patient (41). Family’s level of distress and the parental capacity to contain the child also play a role in the decision-making on the child’s psychiatric hospitalization (42).

Few studies have compared NSSI outpatients with NSSI inpatients. We are aware of only one study that investigated the course of suicidal and non-suicidal self-injury between the two clinical samples, identifying in the hospitalized sample a lower age of onset of such self-injurious phenomena and a significantly higher presence of suicidal ideation and suicidal planning (43). This study considered the current level of care as an inclusion criterion, such that some outpatients might have had previous hospitalizations. Further research, focusing exclusively on groups of self-harm inpatients, has identified an increased suicide risk among those with a previous hospitalization for self-harm (44) and a tendency for repeated self-harm over time, which, in turn, is influenced by traits of emotional dysregulation and internalizing symptoms (45). Moreover, other studies found that both externalizing (e.g., disruptive behavior disorder) and internalizing (e.g., anxiety and depressive disorder) problems were associated with inpatient treatment in adolescents (46, 47).

The current study aimed to further expand knowledge regarding features that characterize young self-harmers who receive different levels of care (i.e., inpatient admission *vs*. outpatient visits), with particular attention on psychopathological, family, and non-suicidal self-harm-related characteristics, as well as suicidality. Specifically, the objectives of the study were as follows: 1) identifying any difference between young NSSI inpatients and young NSSI outpatients in the non-suicidal self-harm-related characteristics (i.e., NSSI methods, NSSI frequency, NSSI method versatility, NSSI function, and number of injured body sites), suicidality (i.e., lifetime suicidal ideation and lifetime suicide attempts), and psychopathological features. We have considered variables that have previously been associated with NSSI, such as alexithymia, emotion dysregulation, psychosocial functioning, externalizing problems, internalizing problems, and total problems. According to the previous findings (41, 43, 48), we supposed that NSSI inpatients reported more lifetime suicidality, greater NSSI severity, and impaired psychosocial functioning than the outpatient group. Moreover, based on studies that focused on hospitalization-related psychopathological factors in psychiatric populations (46, 47, 49), we hypothesized that the inpatient group showed a greater severity of externalizing, internalizing, and total problems than the outpatient group, as well as more severe emotion dysregulation; 2) pinpointing any difference between young NSSI inpatients and young NSSI outpatients in the family interactive-relational variables and investigating any changes in family interaction dynamics in the inpatient and the outpatient groups. Plener and colleagues (2016) highlighted that inpatient treatment for youngs with NSSI was indicated when the environment is detrimental for the recovery in an outpatient setting (41), whereas other studies found that hospitalization in a Child Neuropsychiatric Unit was associated with intrafamily issues (50–52). Despite these findings, we are not aware of studies that focused on specific interactive family dynamics associated with hospitalization in NSSI cases; thus, we did not formulate any hypothesis and we decided to proceed in an explorative way.

## 2 Materials and methods

### 2.1 Participants

**Figure 1:**
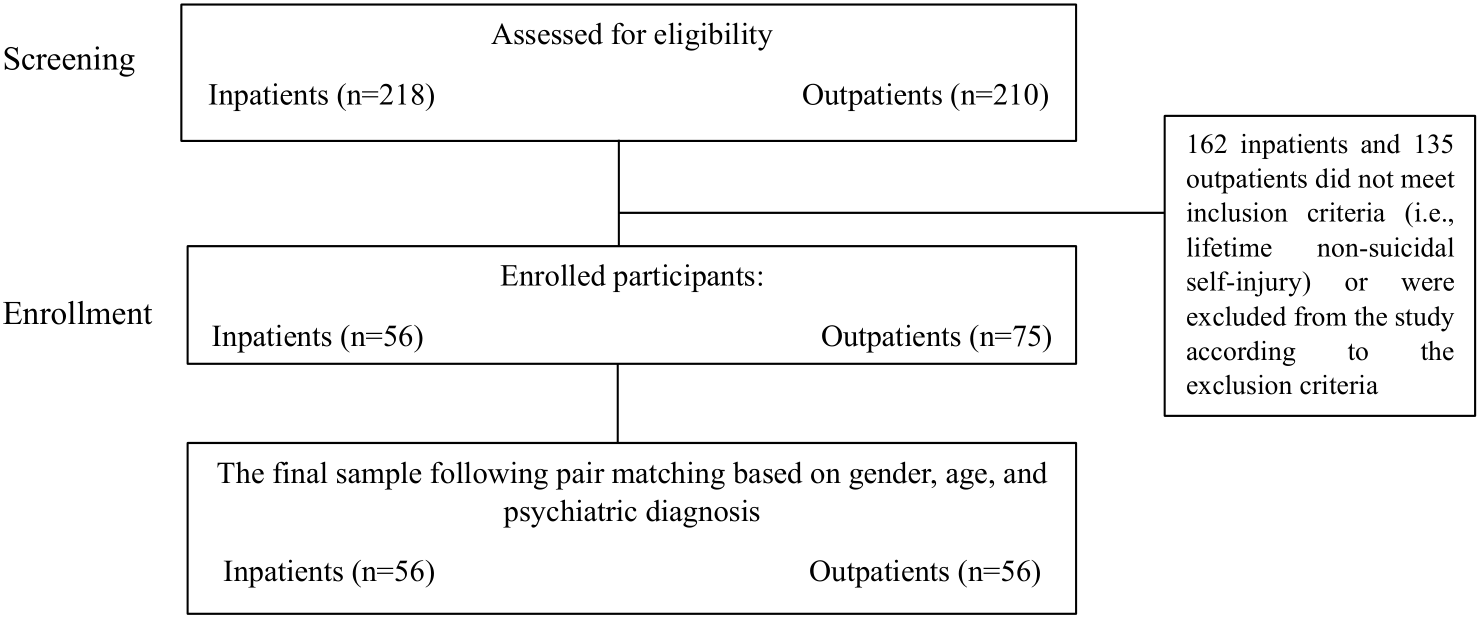
Flow diagram of participants.

The current research is a cross-sectional observational study. It has been screened all inpatients and outpatients who consecutively approached two Child Neuropsychiatry Units in northern Italy in the period between January 2022 and December 2023. We included in the study only patients who reported lifetime non-suicidal self-injury (NSSI) either as a reason for the clinical consultation, hospital admission or emerged from clinical history. For each inpatient included, we matched an oupatient, according to age, gender, and first psychiatric diagnosis (ICD-10) in order to control for these factors. The availability of participants within the clinical settings constrained the sample size. The final sample was composed of fifty-six NSSI inpatients and fifty-six NSSI outpatients. Given the scarcity of male NSSI inpatients who were admitted to the Child Neuropsychiatric Unit, we excluded males in the final sample. Moreover, we excluded outpatients with previous psychiatric hospitalizations, inpatients and outpatients with intellectual disabilities, with autism spectrum disorders and those who did not give informed consent for research participation. Flow diagram of participants is shown in **Figure 1**. The patients’ age range was 11 to 17 years old (*M* = 14.5; *SD* = 1.4). Regarding the distribution of the first psychiatric diagnosis, thirty-two outpatients and thirty-two inpatients had an ongoing diagnosis of mood disorders (F30-39), seventeen outpatients and seventeen inpatients had an ongoing diagnosis of anxiety and somatoform disorders (F40-49), five outpatients and five inpatients had an ongoing diagnosis of behavioral and emotional disorders (F90-98) and two outpatients and two inpatients had an ongoing diagnosis of eating disorders (F50-59). Moreover, forty-five (80%) inpatients and twenty-two (39%) outpatients had at least one psychiatric comorbidity. Forty-three inpatients (77%) were on their first hospitalization for mental issues. All inpatients and 46.9% outpatients were on drug therapy. As regards family characteristics, 10% of outpatients and 26% of inpatients had divorced or separated parents. The parents’ ages ranged from 31 to 57 years old for the mothers (mean age of inpatients’ mothers = 46.6, *SD* = 6.2 and mean age of outpatients’ mothers = 45.6, *SD* = 6.7) and from 33 to 71 years old for the fathers (mean age of inpatients’ fathers = 51.8, *SD* = 7.2 and mean age of outpatients’ fathers = 48.4, *SD* = 7.8). 80% of inpatients and 96% of outpatients had at least one sibling.

### 2.2 Procedure

Data were gathered from patients and their parents during the clinical assessment that occurred in the initial clinical consultation (for outpatients) or in the hospital admission (for inpatients). In the outpatient setting, the clinical evaluation includes a clinical interview with the adolescent patient and their parents conducted by a neuropsychiatrist and a psychologist and the administration of clinical questionnaires (see section ‘Instruments’). In the absence of clinical and family limitations (e.g., family not providing consent for video-recording), multi-problem family situations, etc.), the observation of family interactions is conducted by administering the Lausanne Trilogue Play (LTP) procedure. Regarding the inpatient setting, the clinical assessment was carried out by an interdisciplinary team composed of medical doctors, psychologists, nurses, and educators. The clinical multidisciplinary evaluation includes medical examinations, clinical interviews with the inpatient and their parents, the administration of clinical questionnaires (see section ‘Instruments’), and the observation of family interactions through the use of the Lausanne Trilogue Play (LTP) procedure, administered only to patients without clinical and family limitations, as mentioned above. Both the young patients and their parents provided informed consent for research participation. The current study was conducted in accordance with the Declaration of Helsinki and was approved by the local Ethics Committee (CESC protocol code n° 0044914 of 13 July 2021)

### 2.3 Instruments

The *Youth Self Report* (YSR) (53, 54) is a standardized, widely used self-report instrument completed by adolescents for the assessment of the juvenile’s psycho-behavioral profile (55). It is composed of two parts: the first part assesses adaptive functioning (activities, social, and total competencies); the second part is characterized by 112 items evaluating emotional-behavioral problems according to eight syndrome scales, which were grouped into three broadband scales: internalizing problems (including withdrawn/depressed problems, anxious/depressed problems, somatic complaints); externalizing problems (including aggressive behavior and rule-breaking behavior); and total problems (sum of all items). Moreover, other three syndrome scales were included: social problems, attention problems, and thought problems. According to the score obtained for each scale, behaviors can be evaluated as ‘clinical’, ‘borderline’, or ‘nonclinical’. Furthermore, a Deficient Emotional Self-Regulation (DESR) profile can be obtained by summing the scores of the attention problems, anxious/depressed, and aggressive behavior scales. A score between 180 to 210 suggests poor emotional self-regulation, while 210 or more indicates severe dysregulation (56, 57). According to the purpose of the current study, we considered the scores of three broadband scales (i.e., internalizing, externalizing, and total problems), the subscale ‘activities’ (i.e., youth’s engagement in sport and non-sport related activities), and the DESR profile. Regarding psychometric properties, Frigerio and colleagues (2004) found satisfactory internal consistency of the questionnaire (Cronbach’s alpha values ranging from 0.83 to 0.91) (58).

The *Children’s Global Assessment Scale* (CGAS) (59) is a clinician-reported measure that assesses general psychological and social functioning in children and adolescents ages 4 to 16, but has now been extended to 23 years old (60). Based on the lowest level of functioning in the last month, the clinician evaluates a range of psychological and social aspects and gives a score between 1 and 100. Scores formed ten categories, from ‘constant supervision is required’ (1-10) to ‘superior functioning’ (91-100). Scores higher than 70 indicate good psychosocial functioning. It has been found to have adequate inter-rater reliability (ICC=0.84), satisfactory test-retest reliability (ICC range= 0.69-0.95) and good convergent and discriminant validity (59).

The *Toronto Alexithymia Scale* (TAS-20) (61, 62) is a 20-item, self-administered questionnaire that measures three factors: difficulty in identifying feelings, difficulty in describing feelings, and externally oriented thinking. For the purpose of this study, we calculated the total TAS score by adding the scores from the aforementioned factors. A total score of 60 or higher suggests alexithymia. Several studies have supported the application of this instrument in the juvenile population (63–65). The Italian version of TAS-20 exhibits adequate test-retest reliability and good internal consistency (Cronbach’s α range: 0.52-0.75 for the general population and 0.54-0.82 for clinical samples) (61).

The *Lausanne Trilogue Play* (LTP) procedure (66) is an observational, semi-standardized, distress-free play situation that assesses the triadic family interactions. This procedure involves the adolescent and their parents together, who are requested to plan either the adolescent’s birthday or a daytrip. It requires a specific setting based on a four-part scenario characterized by four subsequent triadic interactive configurations: in the first part (2 + 1), one parent plays with the adolescent while the other parent simply stays nearby; in the second part (2 + 1), the two parents switch roles; in the third part, the parents and the adolescent play together; and in the fourth part (2 + 1), the parents play together whereas the adolescent simply stays nearby. The play situation is video-recorded and subsequently analyzed according to the guidelines provided by the FAAS manual (Family Alliance Assessment Scale 6.3) (67) tailored for adolescents (68). Fifteen interactive-relational variables were evaluated (further details about variables are described in **Table 1**). For each LTP’s part, the variables are evaluated on a three-point Likert scale (1=inappropriate; 2= partially appropriate; 3=appropriate). An overall score for each variable is obtained by summing the scores of the same variable for each part of the LTP. Furthermore, by adding the scores of 15 variables from the same part of the play, a global score for each LTP part is calculated. Scoring was performed by two independent assessors who underwent specific training in the LTP procedure. In the current research, the inter-rater reliability reached a Cohen’s kappa of 0.90, and the overall internal consistency was high (Cronbach’s α=0.91), ranging from 0.90 to 0.94.

**Table 1:** Description of 15 interactive-relational variables assessed during the LTP procedure.

LTP variable	Description
**Posture and gaze**	Ability to create an interactive space facilitating verbal and affective interactions through body posture and gaze
**Inclusion of partners**	Ability to include all partners in the interactive situation
**Role implication**	Ability to respect one’s assigned role in that specific LTP part
**Structure**	Ability to adhere to instructions given at the beginning of the family play in terms of the duration of the play and the execution of all four parts.
**Co-construction**	Ability to sustain a common attentional focus or object of discussion shared by all family members.
**Parental scaffolding**	Parental ability to keep monitoring the child and stimulate him/her appropriately with respect to his/her age and abilities
**Support**	Ability of parents to cooperate and support each other verbally and nonverbally in carrying out the family play
**Conflicts**	Ability of parents not to interfere with each other and not to have conflicting and competitive attitudes
**Interactive mistakes during activities**	Ability of partners to fluidly repair interactive errors without excessive expenditure of energy and time
**Interactive mistakes during transitions**	Ability to manage the transition from one part of the LTP to the other fluidly, with implicit or explicit negotiation of that transition being shared by all partners
**Adolescent’s involvement**	Child’s ability to actively engage in interaction with parents. In the teenager’s age, he or she is able to express his or her ideas clearly, integrate proposals provided by parents, negotiate boundaries, and manage conflicts
**Adolescent’s self-regulation**	Child’s ability to self-regulate during family play, modulating his or her emotional state and staying well predisposed to interact.
**Family warmth**	Family climate is characterized predominantly by a circularity of positive affects and a tendency to empathize even with negative emotional states.
**Validation**	Parents’ ability to recognize their child’s emotional signals, validate and regulate them verbally or nonverbally
**Authenticity**	The affects expressed are spontaneous, appropriate to the interactive situation, corresponding to one’s own expression behavior and to the affective states manifested by the other partners.

### 2.4 Data analysis

Descriptive statistics (i.e., means, standard deviation, and percentage frequencies) were calculated in order to determine the socio-demographic and clinical characteristics of the whole sample and the subgroups of patients divided according to the level of care setting (outpatients *vs*. inpatients). A Chi-square test was applied in order to investigate the differences between outpatients and inpatients in categorical variables in terms of NSSI characteristics, suicidality, and YSR’s psychopathological features, including internalizing, externalizing, and total problems categorized at clinical, borderline, or non-clinical levels. To explore differences between outpatients and inpatients in continuous clinical variables, first, we controlled the assumption of normality and homoscedasticity. The TAS total scores and DESR profile scores satisfied both assumptions, thus the Student’s t-test was applied. Unlikely, CGAS scores did not show homogeneity of variance, thus Welch’s t-test was performed. LTP variable scores did not satisfy the normality assumption, thus the Mann-Whitney test was applied. In order to outline the magnitude of the differences between outpatients and inpatients in the psychopathological variables, effect size indices are reported (i.e., Cohen’s d for continuous normal variables and Cramer’s V for categorical variables). Finally, in order to investigate, separately for the outpatients and inpatients groups, the within-subjects trend of the overall scores among the four parts of the LTP and the scores of each interactive-relational variable among the four parts of the LTP scenario, Friedmann’s test was applied. For this purpose, we selected only families who had completed all four LTP parts. A Durbin-Conover *post-hoc* test was conducted for the pairwise comparison. Due to the exploratory nature of the study, we did not perform a formal power analysis. To handle missing data, we used Available Case Analysis in order to maximize the use of the data without excluding participants due to missing values on some variables. Data analyses were performed using the software Jamovi 2.3.18 (2022).

## 3 Results

### 3.1 NSSI characteristics and suicidality

57.1% outpatients and 65.5% inpatients reported repetitive NSSI (i.e., more than five NSSI acts in the last year). Regarding the NSSI method, scratching was more likely in the inpatient group (χ²(1)=3.91; p=0.048), whereas other NSSI methods (i.e., self-cutting, hitting, head-banging, and burning) did not differ between the inpatient and outpatient groups (see **Table 2**). Average NSSI versatility was 1.16 (SD= 0.42; range=1-3) for the outpatient group and 1.27 (SD=0.52; range=1-3) for the inpatient group, with 85.5% outpatients and 76.4% inpatients using one method, 12.7% outpatients and 20.0% inpatients using two methods, and 1.8% outpatients and 3.6% inpatients using three methods. As regards the NSSI functions, 78.0% outpatients and 61.8% of inpatients used NSSI for reducing negative emotions; 26.8% outpatients and 29.1% inpatients used NSSI for self-punishing; 4.9% outpatients and 10.9% inpatients used NSSI for managing interpersonal difficulties; 4.9% outpatients and 5.5% inpatients used NSSI for inducing positive feelings; 4.9% outpatients and 3.6% inpatients used NSSI for emulation. In 9.8% outpatients and 5.5% inpatients, NSSI was associated with a sense of urgency. 56.9% of outpatients and 63.3% of inpatients reported multiple sites of injury. The NSSI frequency, method versatility, NSSI function, and number of injury sites did not differ between the inpatient and outpatient groups. The inpatient group was more likely to report lifetime suicidal ideation (χ²(1)=12.9; *p*=0.001) and lifetime suicide attempts (χ²(1)=20.0; *p*=0.001) than the outpatient group. However, the difference was still statistically significant for lifetime suicide attempts (χ²(1)=9.84; p=0.002) but not for suicidal ideation (χ²(1)=0.55; p=0.458) when we stratified the data based on the presence of a drug regimen.

**Table 2:** NSSI Characteristics and lifetime suicidality in the inpatient and the outpatient groups.

Variable	Inpatient group N (%)	Outpatient group N (%)	χ² (df)	*p*
NSSI frequency				
>5 acts in the last year	36 (65.5)	32 (57.1)	0.80 (1)	0.369
NSSI Method				
Self-cutting	45 (81.8)	50 (90.9)	1.93 (1)	0.165
Scratching	14 (25.5)	6 (10.9)	3.91 (1)	0.048*
Hitting	5 (9.1)	3 (5.5)	0.53 (1)	0.463
Head-banging	1 (1.8)	0 (0)	1.01 (1)	0.315
Burning	0 (0)	2 (3.6)	2.04 (1)	0.154
Other	4 (7.3)	3 (5.5)	0.15 (1)	0.696
Method versatility				
One method	42 (76.4)	47 (85.5)	1.50 (2)	0.472
Two methods	11 (20.0)	7 (12.7)		
Three methods	3 (3.6)	1 (1.8)		
NSSI function				
Reducing negative emotions	34 (61.8)	32 (78.0)	2.88 (1)	0.090
Self-punishing	16 (29.1)	11 (26.8)	0.05 (1)	0.807
Inducing positive emotions	3 (5.5)	2 (4.9)	0.01 (1)	0.900
Interpersonal difficulties management	6 (10.9)	2 (4.9)	1.12 (1)	0.290
Emulation	2 (3.6)	2 (4.9)	0.09 (1)	0.763
Urgency	3 (5.5)	4 (9.8)	0.64 (1)	0.423
Number of injury sites				
Multiple injury sites	31 (63.3)	29 (56.9)	0.42 (1)	0.514
Lifetime suicidal phenomena				
Lifetime suicidal ideation	49 (87.5)	32 (57.1)	12.9 (1)	<0.001**
Lifetime suicidal attempts	33 (58.9)	10 (17.9)	20.0 (1)	<0.001**

df, degree of freedom; *p<0.05; **p<0.01. *p*-values < 0.05 are bold.

### 3.2 Psychopathological characteristics

In order to determine whether the inpatient and outpatient groups of NSSI adolescents differed in some clinical variables usually associated with NSSI, the Student’s t-test was performed. It has been found that the inpatient group showed a statistically higher total TAS mean score (t(95)=-3.37; *p*=0.001) and a statistically higher DESR mean score (t(103)=-3.16; *p*= 0.002) than the outpatient group, indicating more severe alexithymic traits and more severe difficulties in emotion regulation, respectively. Moreover, the inpatient group exhibited a statistically significant lower CGAS mean score than the outpatient group (t(88)=2.40; *p*=0.018), indicating worse psychosocial functioning. In YSR subscale ‘activities’ 40.5% of outpatients and 59.5% of inpatients reached the clinical level, despite the absence of any statistical significant difference between groups (χ²(2)=4.26; *p*=0.119). Considering the YSR’s broad variables (i.e., internalizing problems, externalizing problems, and total problems), whose severity is evaluated on three categorical levels (i.e., clinical, borderline, and non-clinical), we found a statistically significant association between the level of care (inpatient *vs*. outpatient) and the internalizing (χ²(2)=8.08; *p*=0.018) and externalizing problems (χ²(2)=8.40; *p*=0.015), whereas no statistically significant association has been found for total problems (χ²(2)=5.39; *p*=0.068). Results are shown in **Tables 3** and **4**. By stratifying the data according to the presence of a drug regimen, the difference only remained statistically significant for TAS mean score (t(62)=-3.26; *p*=0.002), DESR mean score (t(66)=-2,30; *p*=0.024), and internalizing problems (χ²(2)=8.58; *p*=0.014).

**Table 3:** Comparison of clinical features between NSSI inpatients and NSSI outpatients.

Clinical variables	Inpatient group M (SD)	Outpatient group M (SD)	Statistical test (df)	*p*	Effect size
**TAS Total Score**	71.3 (8.85)	64.9 (9.73)	Student’s t(95)=-3.37	0.001**	Cohen’s d=-0.685
**DESR score**	211.7 (27.55)	196.1 (22.79)	Student’s t(103)=-3.16	0.002**	Cohen’s d=-0.619
**CGAS score**	47.6 (10.99)	52.3 (7.35)	Welch’s t(88)=2.40	0.018*	Cohen’s d= 0.497

M, Mean; SD, Standard Deviation; df, degree of freedom; *p<0.05; **p<0.01. *p*-values < 0.05 are bold.

**Table 4:** Association between YSR’s clinical variables and the level of care in NSSI adolescents.

YSR variables	Inpatient group			Outpatient group			χ²(df)	*p*	Effect size
	% clinical	% borderline	% non-clinical	% clinical	% borderline	% non-clinical			
**Internalizing problems**	94.4	0.0	5.6	77.4	11.3	11.3	8.08(2)	0.018*	Cramer’s V=0.27
**Externalizing problems**	50.0	18.5	31.5	37.7	5.7	56.6	8.40(2)	0.015*	Cramer’s V=0.28
**Total problems**	85.2	7.4	7.4	66.0	15.1	18.9	5.39(2)	0.068	–

*p<0.05. *p*-values < 0.05 are bold.

### 3.3 The interactive family functioning

For a subgroup of inpatients (N=28) and a subgroup of outpatients (N=19) and their parents, we considered data obtained from the Lausanne Trilogue Play (LTP) procedure. In order to explore the difference in the interactive family dynamics between two groups, the Mann-Whitney test was applied to the global scores of each LTP interactive-relational variable. Higher global scores indicate a higher quality of family interactions. Statistically significant differences between the inpatient and outpatient groups have emerged in the following LTP variables: structure (U=128; *p*=0.009) and interactive mistakes during activities (U=127; *p*=0.009). Descriptive statistics and the Mann-Whitney test are shown in **Table 5**.

**Table 5:** Association between LTP interactive-relational variables and the level of care.

LTP variable	Inpatient group M (SD)	Outpatient group M (SD)	Mann-Whitney U	*p*
Postures and gazes	7.68 (1.91)	7.94 (2.44)	213	0.550
Inclusion of partners	8.29 (2.37)	9.59 (1.94)	165	0.086
Role implication	8.36 (2.11)	9.00 (2.74)	199	0.356
Structure	7.00 (2.05)	5.29 (1.57)	128	0.009*
Co-construction	7.50 (2.47)	7.29 (2.23)	226	0.786
Parental Scaffolding	7.21 (2.48)	7.12 (1.90)	233	0.906
Support	8.54 (2.19)	8.41 (1.80)	238	1.000
Conflicts	10.04 (2.13)	9.47 (2.35)	201	0.382
Adolescent’s involvement	6.54 (2.80)	7.59 (2.15)	176	0.146
Adolescent’s self-regulation	7.04 (2.69)	7.65 (2.74)	207	0.470
Interactive mistakes during activities	8.71 (1.94)	6.88 (2.12)	127	0.009*
Interactive mistakes during transitions	7.86 (2.26)	9.24 (2.54)	157	0.057
Family warmth	7.61 (2.35)	6.88 (2.06)	194	0.304
Validation	6.82 (2.67)	6.88 (2.00)	226	0.777
Authenticity	8.00 (2.42)	8.94 (2.79)	181	0.734

M, Mean; SD, Standard Deviation; *p<0.05. *p*-values < 0.05 are bold.

### 3.4 Within-subjects trend of interactive family dynamics

**Figure 2:**
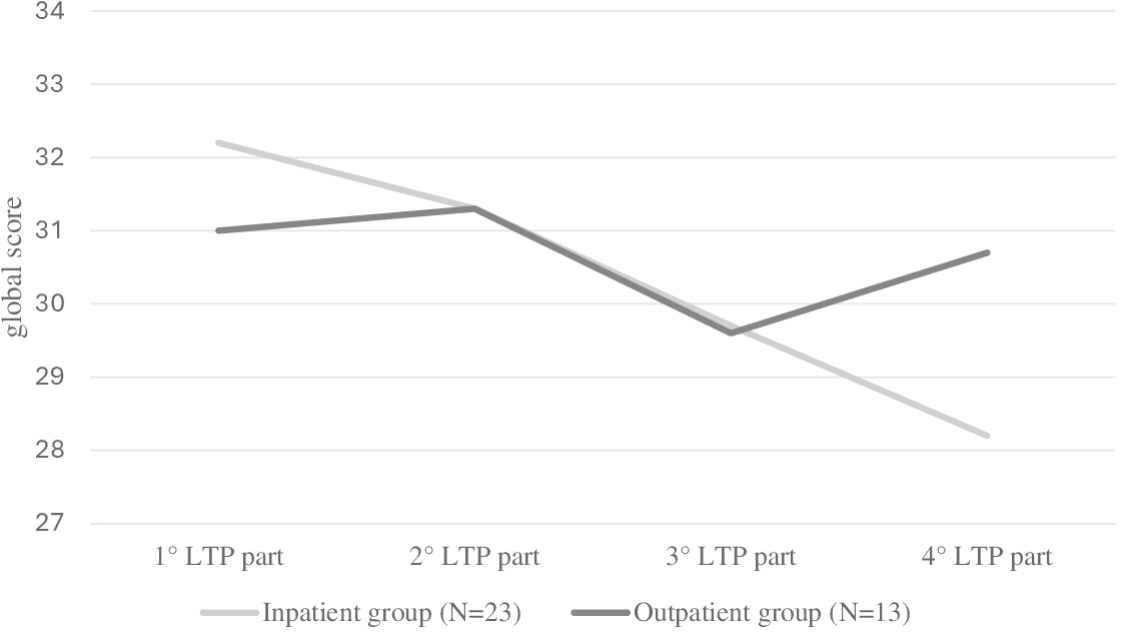
Within-subjects trends in interactive family dynamics in the inpatient group and the outpatient group.

To determine whether the interactive family dynamics changed over the LTP four-part scenario, the Friedmann test was run for the inpatient and the outpatient groups, respectively. We only considered families who had completed all four LTP parts, thus including 13 families for the outpatient group and 23 families for the inpatient group. As regards the outpatient group, there has not been a statistically significant change over the LTP four-part scenario (χ²(3)=2.83; *p*=0.419). On the contrary, for the inpatient group, a statistically significant change from the first LTP part to the fourth LTP part has been found (χ²(3)=14.4; *p*=0.002). No statistically significant difference in any LTP part occurred between the inpatient and the outpatient groups (1° LTP part: U=264; p=0.974; 2° LTP part: U=217; *p*=0.622; 3° LTP part: U=232; *p*=0.730; 4° LTP part: U=167; *p*=0.152). **Figure 2** depicts trends in interactive family dynamics over the LTP four-part scenario for both the inpatient and outpatient groups.

Furthermore, the Friedmann test was performed on each of the LTP interactive-relational variables to determine whether specific LTP variables changed during the four-part scenario for the inpatient and outpatient groups separately. Unlike the outpatient group, in the inpatient group the following LTP variables exhibited a statistically significant change during the LTP four-part scenario: role implication (χ²(3)=10.5; *p*=0.015); parental scaffolding (χ²(3)=11.0; *p*=0.012); conflicts (χ²(3)=11.9; *p*=0.008); adolescent’s involvement (χ²(3)=15.0; *p*=0.002); adolescent’s self-regulation (χ²(3)=18.5; *p*<0.001). Descriptive statistics, the Friedmann Test, and the Durbin-Conover pairwise comparison are described in **Table 6**.

**Table 6:** Within-subjects trends in LTP variables during the four-part scenario.

LTP variables	Group	1° LTP part *M* (*SD*)	2° LTP part *M* (*SD*)	3° LTP part *M* (*SD*)	4° LTP part *M* (*SD*)	χ² (df)	*p*	Durbin-Conover pairwise comparison
LTP total score	IP	32.2 (5.74)	31.3 (6.52)	29.7 (6.38)	28.2 (6.10)	14.4 (3)	0.002*	1° LTP part > 3° LTP part* 1° LTP part > 4° LTP part*** 2° LTP part > 4° LTP part*
	OP	31.0 (4.02)	31.3 (4.07)	29.6 (6.45)	30.7 (7.48)	2.83 (3)	0.419	–
Postures and gazes	IP	2.09 (0.42)	2.00 (0.52)	2.04 (0.37)	1.96 (0.37)	1.14 (3)	0.767	–
	OP	1.92 (0.76)	2.00 (0.58)	2.08 (0.64)	2.46 (0.77)	5.06 (3)	0.167	–
Inclusion of partners	IP	2.17 (0.65)	2.13 (0.55)	2.30 (0.47)	2.09 (0.73)	3.12 (3)	0.373	–
	OP	2.38 (0.87)	2.54 (0.66)	2.46 (0.66)	2.31 (0.85)	1.96 (3)	0.581	–
Role implication	IP	2.39 (0.58)	2.13 (0.63)	2.17 (0.65)	1.91 (0.79)	10.5 (3)	0.015*	1° LTP part > 2° LTP part* 1° LTP part > 4° LTP part***
	OP	2.85 (0.38)	2.54 (0.78)	2.25 (0.87)	2.15 (1.92)	5.86 (3)	0.119	–
Structure	IP	2.00 (0.85)	1.96 (0.82)	1.61 (0.72)	1.74 (0.81)	2.02 (3)	0.568	–
	OP	1.15 (0.38)	1.31 (0.48)	1.46 (0.78)	1.69 (0.95)	3.66 (3)	0.300	–
Co-construction	IP	2.00 (0.80)	2.04 (0.82)	1.74 (0.75)	2.04 (0.71)	4.53 (3)	0.209	–
	OP	1.69 (0.48)	2.00 (0.70)	1.69 (0.75)	2.15 (0.69)	5.89 (3)	0.117	–
Parental Scaffolding	IP	2.09 (0.67)	1.91 (0.79)	1.70 (0.70)	1.61 (0.78)	11.0 (3)	0.012*	1° LTP part > 3° LTP part* 1° LTP part > 4° LTP part** 2° LTP part > 4° LTP part*
	OP	1.77 (0.60)	1.85 (0.55)	1.62 (0.65)	1.92 (0.95)	2.00 (3)	0.572	–
Support	IP	2.26 (0.69)	2.17 (0.58)	2.26 (0.69)	2.13 (0.55)	1.72 (3)	0.632	–
	OP	2.46 (0.52)	2.38 (0.65)	2.31 (0.63)	2.08 (0.86)	1.78 (3)	0.620	–
Conflicts	IP	2.78 (0.52)	2.65 (0.57)	2.70 (0.63)	2.39 (0.58)	11.9 (3)	0.008**	1° LTP part > 4° LTP part*** 2° LTP part > 4° LTP part** 3° LTP part > 4° LTP part**
	OP	2.69 (0.63)	2.46 (0.66)	2.62 (0.65)	2.46 (0.66)	3.24 (3)	0.356	–
Adolescent’s involvement	IP	1.91 (0.85)	1.83 (0.78)	1.61 (0.72)	1.43 (0.66)	15.0 (3)	0.002**	1° LTP part > 3° LTP part* 1° LTP part > 4° LTP part*** 2° LTP part > 4° LTP part**
	OP	1.85 (0.38)	2.00 (0.40)	1.69 (0.63)	1.92 (0.95)	2.04 (3)	0.565	–
Adolescent’s self-regulation	IP	2.09 (0.67)	2.00 (0.74)	1.70 (0.70)	1.57 (0.73)	18.5 (3)	<0.001***	1° LTP part > 3° LTP part** 1° LTP part > 4° LTP part*** 2° LTP part > 3° LTP part* 2° LTP part > 4° LTP part***
	OP	2.00 (0.71)	2.00 (0.82)	1.77 (0.83)	1.77 (0.83)	3.60 (3)	0.308	–
Interactive mistakes during activities	IP	2.39 (0.66)	2.26 (0.75)	2.30 (0.63)	2.00 (0.67)	7.27 (3)	0.064	–
	OP	1.85 (0.55)	1.85 (0.69)	1.92 (0.64)	1.77 (0.72)	0.60 (3)	0.896	–
Interactive mistakes during transitions	IP	2.17 (0.58)	2.00 (0.60)	2.04 (0.77)	1.96 (0.71)	4.06 (3)	0.255	–
	OP	2.69 (0.48)	2.38 (0.65)	2.31 (0.85)	2.31 (0.75)	4.42 (3)	0.219	–
Family warmth	IP	2.04 (0.56)	2.13 (0.69)	1.87 (0.76)	1.91 (0.51)	5.24 (3)	0.155	–
	OP	1.69 (0.63)	1.69 (0.75)	1.69 (0.75)	1.77 (0.72)	0.281 (3)	0.964	–
Validation	IP	1.87 (0.76)	1.87 (0.81)	1.70 (0.76)	1.52 (0.66)	5.73 (3)	0.125	–
	OP	1.62 (0.77)	1.85 (0.69)	1.54 (0.52)	1.77 (0.72)	2.44 (3)	0.487	–
Authenticity	IP	2.17 (0.58)	2.17 (0.65)	2.00 (0.74)	1.91 (0.60)	6.38 (3)	0.094	–
	OP	2.38 (0.65)	2.46 (0.66)	2.38 (0.87)	2.15 (0.80)	3.73 (3)	0.292	–

M, Mean; SD, Standard Deviation; df, degree of freedom; IP, Inpatients; OP, Outpatients; *p<0.05; **p<0.01; ***p<0.001. *p*-values < 0.05 are bold.

## 4 Discussion

The main purpose of the current study was to investigate psychopathological, family, and self-harm- related features that distinguish young NSSI inpatients from young non-suicidal self-injurers who received outpatient treatment. According to Glenn and colleagues (2017) (43), we found that inpatient self-harmers reported higher rates of lifetime suicidal ideation and lifetime suicidal attempts than the outpatient group. Differently from Glenn and colleagues (2017) (43), we did not include outpatients with a previous history of psychiatric hospitalization; thus, this highlighted that the two groups differ from the intensity of level of care received in their lifetime for mental health issues. Our findings were consistent with the fact that hospitalization is often the treatment of choice for individuals with high suicidal risk (69). Contrary to our expectations, the inpatient group did not show more severe NSSI in terms of method versatility and NSSI frequency, even though the inpatient group reported higher rates of scratching, which was associated in the previous studies with a more severe risk profile (70) and with interpersonal conflict management and regulation of negative emotions (71). Consistently, we found that the inpatient group showed a more severe emotion dysregulation profile, higher alexithymic traits, and worse psychosocial functioning than the outpatient group. In addition, clinical levels of externalizing and internalizing problems were more associated with the inpatient treatment. Overall, these findings supported a more severe global clinical picture of self-harmers who were required to have a more intensive level of care. Although the association between the level of care and certain variables (such as lifetime suicidal ideation, externalizing problems, and psychosocial functioning) was lessened when a drug regimen was taken into account, the degree of underlying psychopathology — rather than the severity of the NSSI characteristics — seems to be what distinguishes the inpatient sample from the outpatient sample. This suggests that NSSI should not be viewed as a symptom in and of itself, but rather as an expression of a larger psychopathological picture that requires thorough investigation.

The evaluation of interactive family dynamics through the LTP procedure highlighted that the inpatient group showed better scores in the variables ‘structure’ and ‘interactive mistakes during activities’ than the outpatient group. In fact, hospitalization at a Child Neuropsychiatric Unit requires that all family members define new roles and responsibilities together, adjust their daily routines and adapt themselves to the strict rules and routines imposed by the admission (72–74). As a result, our findings may be understood in view of the fact that the inpatient group’s families have become accustomed to changing their activities as part of an adaptation process in response to external circumstances (e.g., the hospital admission) and strictly adhering to explicit hospital rules. Moreover, these findings emphasize the role of hospitalization as a structural framework within which the young patient and family can get care and containment at a time when the family setting alone cannot provide a safe and conflict-free environment for the child’s growth.

In addition, unlike the outpatient group’s families, the inpatient group’s families showed a decrease in the overall interactive-relational performance during the course of the LTP four-part scenario, reaching the global lowest score in the fourth part, in which the play situation is mostly managed by the co-parenting system. This decreasing trend has also been found in some of the specific LTP interactive-relational variables, such as ‘role implication’, ‘parental scaffolding’, ‘conflicts’, ‘adolescent’s involvement’, and ‘adolescent’s self-regulation’. A two-way process emerged: both the parents’ ability to monitor and stimulate appropriately the child and the child’s ability to self-regulate and appropriately involve in family interactions collapsed into the fourth part of the family play situation, when the co-parenting system was left to manage the family interactions and the adolescent simply had to stay nearby. At this stage, conflicting and interfering parental interactions peaked.

A further contribution of our research consists of showing that the adolescent’s self-dysregulation is not a fixed dimension but changes across the LTP four-part scenario, according to different triadic configurations. It reached its maximum peak when the adolescent should act as a simple observer within a family context characterized by lowered parental scaffolding and high conflicts. These findings were consistent with the biopsychosocial model of mental health (75), which emphasizes the role of the family and social environment for the child’s development (28). Previous studies highlighted the role of the co-parenting system in the child’s mental health issues (76, 77). In fact, it has been demonstrated that families of hospitalized children were often characterized by high interparental conflicts (50, 51) and low parental capacity to contain the child (42), which, in turn, were associated with a low child’s ability to regulate own emotions (78, 79). To the best of our knowledge, the current study is the first to report findings on the changes in interactive family dynamics using an observational semi-standardized tool in hospitalized adolescents with self-harming, while also disentangling family, psychopathological, and NSSI-related features from an adolescent NSSI outpatient sample. Overall, our results highlighted the relevance of evaluating the co-parental system during the hospitalization process of young self-harmers using multi-informant and multi-method instruments, given that the quality of interparental interactions may have a role in the child’s self-regulation.

The current study presents some limitations. First, because of the current study’s small sample size and the sample enrollment occurring only at two Child Neuropsychiatry Units in northern Italy, it is necessary to be cautious in generalizing the results. Particularly, we were able to obtain data on interactive family dynamics just for a small subgroup of self-harmers due to the presence of family limitations (e.g., problematic family situations). Future research involving multisite sampling across different areas of Italy are needed. Moreover, all patients were females; thus, it did not allow us to extend the findings to the male population of self-harmers. Further studies should enroll larger samples, including male self-harmers, to conduct subgroup analysis according to gender and increase the external validity of study results. Given the cross-sectional nature of this study, it poses a challenge to determine whether the findings pertaining to clinical data and family functioning also serve as a predictor for the type of psychiatric treatment that follows. Hence, prospective longitudinal studies are needed for further research in this field. Although similar inclusion and exclusion criteria between groups have been chosen (with the exception of the variable ‘previous psychiatric hospitalizations’), it is possible that other sources of selection bias acted during the sample enrollment, given the several factors that influence the choice of the outpatient level of care *vs*. hospitalization. We only controlled the effect of gender, age, and psychiatric diagnosis on results by using pair matching without considering other potential confounding variables such as psychiatric comorbidity, physical illness, psychiatric familiarity, intrafamily issues, and traumatic events, which should be included in future research. The absence of assessment of the influence of other family factors (such as the number of family members, family income, and family beliefs) on interactive family dynamics is an additional limitation of the study. Finally, we did not consider other environmental factors such as negative peer relationships or school-related problems, which could contribute to mental health issues. The multi-informant and multi-method approach used in this study allows to overcome limitations connected to the exclusive use of self-report instruments, which may be influenced by individual biases such as social desirability or failure to understand questions. In conclusion, disentangling throughout multi-informant and multimethod evaluation the psychopathological, family, and NSSI-related characteristics of young patients with self-harm who required different levels of care (hospitalization *vs*. outpatient visits) is a promising research field with high clinical relevance for the advancement of the diagnostic and therapeutic process that characterizes daily clinical practice.

## 5 Conclusion

The current study highlighted that NSSI inpatients showed a more severe global clinical picture, confirming the need for a more severe level of care for this group. In fact, hospitalization represents a structural framework that works as a source of containment and support for young patients and their families that show greater difficulties for sustaining the child’s development at a certain time. The absence of group difference in NSSI-related characteristics suggested that NSSI should be considered an expression of a larger psychopathological picture, in which contextual factors such as family dynamics could play a role. Therefore, the need for a multi-informant and multi-method clinical assessment, which includes the investigation of environmental factors (e.g., family system and co-parenting system), is highlighted. Future research should further investigate the characteristics of family relationships of NSSI inpatients, using longitudinal studies that allow us to understand the changes of family dynamics over time.

## Data Availability

The raw data supporting the conclusions of this article will be made available by the authors, without undue reservation.
